# Effects of Intermediates between Vitamins K_2_ and K_3_ on Mammalian DNA Polymerase Inhibition and Anti-Inflammatory Activity

**DOI:** 10.3390/ijms12021115

**Published:** 2011-02-10

**Authors:** Yoshiyuki Mizushina, Jun Maeda, Yasuhiro Irino, Masayuki Nishida, Shin Nishiumi, Yasuyuki Kondo, Kazuyuki Nishio, Kouji Kuramochi, Kazunori Tsubaki, Isoko Kuriyama, Takeshi Azuma, Hiromi Yoshida, Masaru Yoshida

**Affiliations:** 1 Laboratory of Food & Nutritional Sciences, Department of Nutritional Science, Kobe-Gakuin University, Nishi-ku, Kobe, Hyogo 651-2180, Japan; 2 Cooperative Research Center of Life Sciences, Kobe-Gakuin University, Chuo-ku, Kobe, Hyogo 650-8586, Japan; 3 Division of Gastroenterology, Department of Internal Medicine, Graduate School of Medicine, Kobe University, Chuo-ku, Kobe, Hyogo 650-0017, Japan; 4 The Integrated Center for Mass Spectrometry, Graduate School of Medicine, Kobe University, Chuo-ku, Kobe, Hyogo 650-0017, Japan; 5 Graduate School of Life and Environmental Science, Kyoto Prefectural University, Sakyo-ku, Kyoto 606-8522, Japan; 6 Division of Metabolomics Research, Graduate School of Medicine, Kobe University, Chuo-ku, Kobe, Hyogo 650-0017, Japan

**Keywords:** vitamin K, MK-2, DNA polymerase λ, enzyme inhibitor, anti-inflammation

## Abstract

Previously, we reported that vitamin K_3_ (VK_3_), but not VK_1_ or VK_2_ (=MK-4), inhibits the activity of human DNA polymerase γ (pol γ). In this study, we chemically synthesized three intermediate compounds between VK_2_ and VK_3_, namely MK-3, MK-2 and MK-1, and investigated the inhibitory effects of all five compounds on the activity of mammalian pols. Among these compounds, MK-2 was the strongest inhibitor of mammalian pols α, κ and λ, which belong to the B, Y and X families of pols, respectively; whereas VK_3_ was the strongest inhibitor of human pol γ, an A-family pol. MK-2 potently inhibited the activity of all animal species of pol tested, and its inhibitory effect on pol λ activity was the strongest with an IC_50_ value of 24.6 μM. However, MK-2 did not affect the activity of plant or prokaryotic pols, or that of other DNA metabolic enzymes such as primase of pol α, RNA polymerase, polynucleotide kinase or deoxyribonuclease I. Because we previously found a positive relationship between pol λ inhibition and anti-inflammatory action, we examined whether these compounds could inhibit inflammatory responses. Among the five compounds tested, MK-2 caused the greatest reduction in 12-*O*-tetradecanoylphorbol-13-acetate (TPA)-induced acute inflammation in mouse ear. In addition, in a cell culture system using mouse macrophages, MK-2 displayed the strongest suppression of the production of tumor necrosis factor (TNF)-α induced by lipopolysaccharide (LPS). Moreover, MK-2 was found to inhibit the action of nuclear factor (NF)-κB. In an *in vivo* mouse model of LPS-evoked acute inflammation, intraperitoneal injection of MK-2 in mice led to suppression of TNF-α production in serum. In conclusion, this study has identified VK_2_ and VK_3_ intermediates, such as MK-2, that are promising anti-inflammatory candidates.

## 1. Introduction

The human genome encodes at least 15 DNA polymerases (pols) that conduct cellular DNA synthesis [1,2]. Eukaryotic cells contain three replicative pols (α, δ and ɛ), 1 mitochondrial pol (γ), and at least 11 non-replicative pols (β, ζ, η, θ, ι, κ, λ, μ, ν, terminal deoxynucleotidyl transferase (TdT) and REV1) [3,4]. Pols have a highly conserved structure, which means that their overall catalytic subunits show little variance among species. Enzymes with conserved structures usually perform important cellular functions, the maintenance of which provides evolutionary advantages. On the basis of sequence homology, eukaryotic pols can be divided into four main families, termed A, B, X and Y [4]. Family A includes mitochondrial pol γ, as well as pols θ and ν. Family B includes three replicative pols (α, δ and ɛ) and pol ζ. Family X comprises pols β, λ and μ, as well as TdT; and lastly, family Y includes pols η, ι and κ, in addition to REV1. We have been studying selective inhibitors of each pol derived from natural products including food materials and nutrients for more than 15 years [5,6]. We have found that vitamin K_3_ (VK_3_), but not VK_1_ or VK_2_, is a potent and specific inhibitor of human pol γ [7–10].

**Figure 1.**
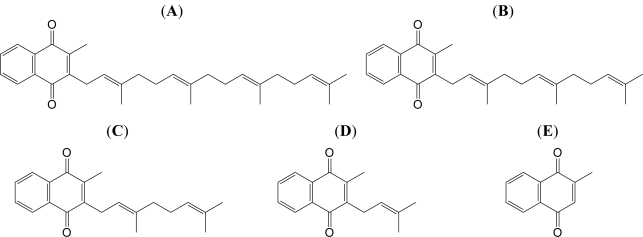
Structures of the VK_2_ and VK_3_ intermediates. (**A**) MK-4 (VK_2_), (**B**) MK-3, (**C**) MK-2, (**D**) MK-1, and (**E**) VK_3_.

VK comprises a family of structurally similar, fat-soluble 2-methyl-1,4-naphthoquinones, including phylloquinone (VK_1_), menaquinone (VK_2_), and menadione (VK_3_). 1,4-Naphthoquinones form a family of compounds characterized by a naphthalene ring that contains two carbonyl moieties at positions 1 and 4, and that, in the case of VK, is substituted at positions 2 and 3 (Figure 1). All members of the VK family possess an identical naphthoquinone skeleton with various side chains that distinguish them. VK_1_ and VK_2_ differ only in the prosthetic group at position 3. VK_1_ possesses a phytyl group (partially saturated poly-isoprenoid group) at position 3, whereas VK_2_ possesses a repeating unsaturated trans-poly-isoprenyl group. The IUPAC-IUB Commission on Biochemical Nomenclature abbreviates VK_2_ as “MK-*n*”, where “*n*” signifies the number of unsaturated isoprene units that compose the side chain at the 3-position. The side chain of MK-*n* can vary in length from C5 (*n* = 1) to C65 (*n* = 13); for example, menaquinone 4 (MK-4) could also be written as K2(20). MK-4 (=VK_2_) has three isoprene units plus the first saturated group beginning at position 3, totaling four (Figure 1A). The most common form of VK in animals is VK_2_ in its MK-4 structure, which is produced by intestinal bacteria from exogenous naphthoquinones and transformed endogenously in our own cells [11]. VK_3_ possesses a much simpler structure, with no aliphatic chain prosthetic group at position 3 (Figure 1E). Although VK_3_ is considered a synthetic analogue, Billeter *et al.* found that VK_1_ can be cleaved to form VK_3_ by bacteria in the intestine [12]. After absorption, VK_3_ is thought to become alkylated into biologically active isoprenylated VK_2_ [13]. However, VK_3_ cannot exert all of the functions of natural VK, a finding that is ascribed to its limited transformation into the fat-soluble vitamin forms [14,15].

In our pol inhibitor studies, we have found that pol λ-selective inhibitors, such as curcumin derivatives [16–18], have 12-*O*-tetradecanoylphorbol-13-acetate (TPA)-induced anti-inflammatory activity [19–21]. Although tumor promoters are classified as compounds that promote tumor formation [22], they also cause inflammation and are commonly used as artificial inducers of inflammation in order to screen for anti-inflammatory agents [23]. Tumor promoter-induced inflammation can be distinguished from acute inflammation, which is exudative and is accompanied by fibroblast proliferation and granulation. The tumor promoter TPA is frequently used to search for new types of anti-inflammatory compound. TPA not only causes inflammation, but also influences mammalian cell growth [24], suggesting that the molecular basis of the inflammation stems from pol reactions related to cell proliferation. This relationship, however, needs to be investigated more closely.

In many inflammatory responses, activation of nuclear factor (NF)-κB is the rate-limiting step of the inflammatory mechanism [25]. The five members of the mammalian NF-κB family, namely p65 (RelA), RelB, c-Rel, p50/p105 (NF-κB1) and p52/p100 (NF-κB2), exist in unstimulated cells as homodimers or heterodimers bound to proteins of the IκB family [26]. The binding of NF-κB to IκB prevents NF-κB from translocating to the nucleus, thereby maintaining NF-κB in an inactive state. NF-κB proteins are characterized by the presence of a conserved 300-amino-acid Rel homology domain located in the *N*-terminus of the protein, and this domain is responsible for dimerization with NF-κB, interaction with IκB, and binding to DNA [26]. The translocated NF-κB proteins work as transcription factors and regulate the expression of various genes that encode proinflammatory cytokines such as tumor necrosis factor (TNF)-α and interleukin (IL)-12, which have been shown to play important roles in sustained inflammatory responses [27–29].

In this study, we focused on compounds that are intermediates between VK_2_ (MK-4) and VK_3_, and chemically synthesized three such compounds (MK-3, MK-2 and MK-1) as derivatives of VK_2_ and VK_3_ (Figure 1B–D). We investigated the inhibitory effects of these five compounds on mammalian pol activity and inflammatory responses both *in vitro* and *in vivo*. We found that some of the VK_2_ and VK_3_ intermediates suppress NF-κB activation induced by lipopolysaccharide (LPS) in mouse macrophage cells. Moreover, we also demonstrated that these compounds exert inhibitory effects against TNF-α production in an animal model of LPS-induced acute inflammation. The goal of this study is clarified whether VK_2_ and VK_3_ intermediates could be a potent chemopreventive agent against inflammation.

## 2. Results

### 2.1. Effect of the VK_2_ and VK_3_ Intermediates on Mammalian Pol Activity

**Figure 2.**
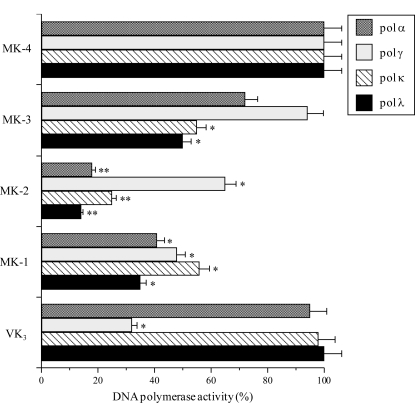
Inhibitory effects of the VK_2_ and VK_3_ intermediates on the activity of mammalian pols. Each compound (50 μM) was incubated with calf pol α (B-family pol), human pol γ (A-family pol), human pol κ (Y-family pol) and human pol λ (X-family pol) (0.05 units each). Pol activity in the absence of the compound was taken as 100%, and the relative activity is shown. Data are shown as the mean ± SE (*n* = 3). ** *P* < 0.01 and * *P* < 0.05 *versus* controls.

Initially, we investigated the *in vitro* biochemical action of VK_2_ (MK-4), VK_3_ and their chemically synthesized derivatives, MK-3, MK-2 and MK-1. The inhibition of four mammalian pols, namely calf pol α, human pol γ, human pol κ and human pol λ, by 50 μM of each compound was investigated. Pols α, γ, κ and λ were used as representatives of the B, A, Y and X families of pols, respectively [1–3]. As shown in Figure 2, MK-3, MK-2 and MK-1, which are intermediates between VK_2_ and VK_3_, inhibited the activity of mammalian pols α, κ and λ, whereas MK-4 (VK_2_) had no effect on pol activity. VK_3_ selectively inhibited pol γ among the mammalian pols tested. The inhibitory effect of these compounds on pols α, κ and λ ranked as follows: MK-2 > MK-1 > MK-3 > MK-4 = VK_3_; and the inhibitory effect of these compounds on pol γ ranked as follows: VK_3_ > MK-1 > MK-2 > MK-3 > MK-4. The IC_50_ values of MK-2 against pols α, γ, κ and λ were 27.6, 68.8, 35.3 and 24.6 μM, respectively. When activated DNA (*i.e.*, bovine deoxyribonuclease I-treated DNA) and dNTP were used as the DNA template-primer and nucleotide substrate instead of synthesized DNA [poly(dA)/oligo(dT)_18_ (A/T = 2/1)] and dTTP, respectively, the inhibitory effects of these compounds did not change (data not shown).

### 2.2. Effects of MK-2 on Pols and Other DNA Metabolic Enzymes

Among the five compounds investigated, MK-2 displayed the strongest inhibitory effect on mammalian pols (Figure 2) and was therefore the focus of this section. As described briefly in the introduction, we have succeeded in obtaining ten eukaryotic pol species including pols α, β, γ, δ, ɛ, ι, η, κ and λ, and TdT; however, pols ζ, θ, μ and ν, and REV1 are not yet available (Table 1). Currently, eukaryotes are thought to express at least 15 species of pols [1,2], and we are still in an era when most pols are very difficult to obtain in their purified form in a laboratory. Table 1 shows the inhibitory effect (IC_50_ value) of MK-2 against various pol species including the ten eukaryotic pols that can be obtained. This compound inhibited the activity of all of the pols from mammals, and 50% inhibition of the A, B, X and Y families of pols was observed at a dose of 68.8, 27.6–29.1, 24.6–31.0 and 35.3–39.0 μM, respectively; therefore, the strength of the inhibitory effect of MK-2 on mammalian pol families can be ranked as follows: B-family pols = X-family pols > Y-family pols > A-family pol. MK-2 showed the strongest inhibition of pol λ activity among the pols investigated, with an IC_50_ value of 24.6 μM. This compound also suppressed the activity of the animal pols from fish (cherry salmon) and insect (fruit fly) at almost the same concentrations as it inhibited the activity of mammalian pols.

By contrast, MK-2 had no effect on plant (cauliflower) pol α or prokaryotic pols, such as *E. coli* pol I, *Taq* pol or T4 pol (Table 1). The three-dimensional structures of eukaryotic pols are likely to differ greatly from those of prokaryotic pols. MK-2 did not inhibit the activity of other DNA metabolic enzymes, such as calf primase pol α, T7 RNA polymerase, T4 polynucleotide kinase, or bovine deoxyribonuclease I. These results suggest that MK-2 may be a selective inhibitor of animal pols, especially the B and X families of pols containing pol λ.

To test whether MK-2 is an intercalating agent that distorts DNA and subsequently inhibits enzyme activity, we measured the thermal transition of DNA in the presence or absence of this compound. The thermal transition profile of DNA was the same with or without the compound (data not shown). Therefore, the inhibition of pols by MK-2 is not due to DNA distortion, but seems to be due to a direct effect of this compound on the enzymes themselves.

### 2.3. Effect of the VK_2_ and VK_3_ Intermediates on TPA-Induced Anti-Inflammatory Activity

**Figure 3.**
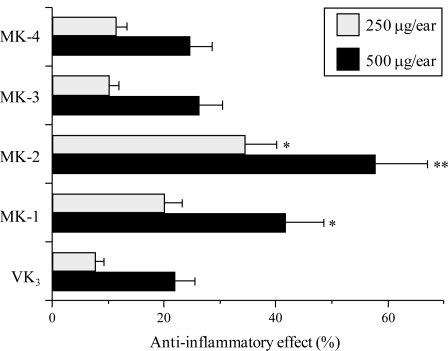
Anti-inflammatory activity of the VK_2_ and VK_3_ intermediates toward TPA-induced edema on mouse ear. Each compound (250 μg, gray bar; and 500 μg, black bar) was applied individually to one ear of a mouse, and after 30 min TPA (0.5 μg) was applied to both ears. Edema was evaluated after 7 h. The inhibitory effect is expressed as the percentage of edema. Data are shown as the means ± SE (*n* = 6). ** *P* < 0.01 and * *P* < 0.05 *versus* controls.

In previous a pol inhibitor study, we found that there is a relationship between pol λ inhibitors and TPA-induced acute anti-inflammatory activity [6,19,20]. Thus, using the mouse ear inflammatory test, we examined the anti-inflammatory activity of the intermediates between VK_2_ and VK_3_. Application of TPA (0.5 μg) to the mouse ear induced edema, resulting in a 241% increase in the weight of the ear disk 7 h after application. As shown in Figure 3, pretreatment with MK-2 and MK-1 suppressed inflammation, and the effect of MK-2 was stronger than that of MK-1. By contrast, MK-4, MK-3 and VK_3_ had a weak effect on the level of inflammation. Therefore, the anti-inflammatory effect of these compounds correlated with their inhibitory effect on mammalian pols including pol λ, which was strongly inhibited by MK-2 (Figure 2). These results suggest that inhibition of pol λ activity has a positive correlation with the anti-inflammatory activity observed.

### 2.4. Inhibitory Effect of the VK_2_ and VK_3_ Intermediates on the LPS-Induced Inflammatory Response in Cultured Macrophage Cells

**Figure 4.**
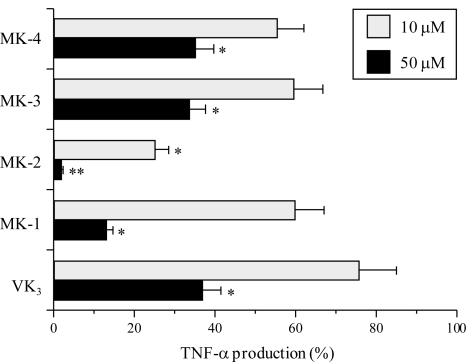
Inhibitory effects of the VK_2_ and VK_3_ intermediates on LPS-induced production of TNF-α in the mouse macrophage cell line RAW264.7. These cells were pretreated with 10 and 50 μM of each compound as a vehicle control (TNF-α level, 52 pg/mL) for 30 min and then treated with 100 ng/mL LPS for 24 h (LPS-evoked TNF-α level, 448 pg/mL). The TNF-α concentration in the cell medium was measured by ELISA. The relative effect in the absence of the compound was taken as 100%. Data are shown as the mean ±SE (*n* = 4). ** *P* < 0.01 and * *P* < 0.05 *versus* controls.

Next, we investigated whether the intermediates between VK_2_ and VK_3_ can inhibit both the reduction in TNF-α production and the nuclear translocation of NF-κB p65 induced by LPS stimulation in cultured mouse macrophage RAW264.7 cells. The inflammatory cytokine TNF-α activates the NF-κB signaling pathway by binding to the TNF-α receptor (TNFR) and thereby initiates an inflammatory response, resulting in various inflammatory diseases [30]. In RAW264.7 cells, no compound showed cytotoxicity at 50 μM, because the LD_50_ values of these five compounds were >100 μM. As shown in Figure 4, MK-2 at 10 and 50 μM, and MK-1 at 50 μM significantly suppressed the LPS-stimulated production of TNF-α, although MK-4, MK-3 and VK_3_ displayed a moderate inhibitory effect on TNF-α production. The inhibitory effects of MK-2 and MK-1 were, respectively, the first and second strongest among these compounds. The effect of the compounds on the suppression of LPS-evoked TNF-α production showed almost the same tendency as their inhibitory effect on mammalian pols including pol λ. These results suggest that VK_2_ and VK_3_ intermediates, such as MK-2, inhibit the activities of mammalian pols, and then prevent the TNF-α production in the LPS-induced macrophages, but not affect the cell growth.

**Figure 5.**
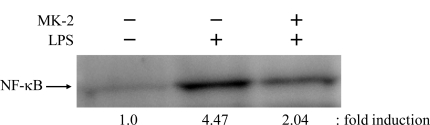
Inhibitory effects of MK-2 on nuclear translocation of NF-κB in RAW264.7 cells. The cells were incubated with 10 μM MK-2 (+) or DMSO (−), as a vehicle control for 30 min, and then with 100 ng/mL LPS for 30 min. Nuclear proteins were prepared from the cells and subjected to Western blot analysis for evaluation of the nuclear translocation of NF-κB p65. The intensity of each band was analyzed, and the values relative to treatment without LPS (negative control, taken as 1.0-fold) are represented at the lower edge of the image.

NF-κB is known to be the rate-controlling factor for inflammatory responses. We therefore examined the inhibitory effect of MK-2 on the LPS-induced nuclear translocation of NF-κB in RAW264.7 cells (Figure 5). By Western blot analysis, it was revealed that the amount of NF-κB nuclear translocation in RAW264.7 cells was 4.47-fold greater after LPS treatment, and that 10 μM MK-2 was sufficient to inhibit the LPS-stimulated nuclear translocation of NF-κB to 2.04-fold. These results demonstrate that this compound can strongly suppress the nuclear translocation of NF-κB by inhibiting the production of TNF-α. The effects of MK-2 on the molecular mechanism of anti-inflammation will be addressed in future studies.

### 2.5. Inhibitory Effect of MK-2 on LPS-Induced Inflammation *in Vivo*

**Figure 6.**
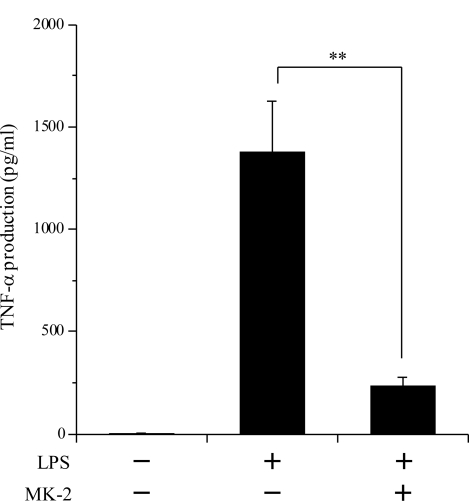
The inhibitory activity of MK-2 against LPS-induced inflammation *in vivo*. Female C57BL/6 mice were intraperitoneally injected with MK-2 at 100 mg/kg BW or corn oil as a vehicle control. After 30 min, the mice were intraperitoneally injected with LPS at 250 μg/kg BW or saline as a vehicle control. One hour after the LPS injection, the mice were killed, and the TNF-α level in serum was measured using ELISA. Treatment with corn oil and LPS was a positive control (TNF-α level, 1376 pg/mL), and that with corn oil and saline was a negative control (TNF-α level, 4.2 pg/mL). Data are shown as the mean ±SE (*n* = 5). ** *P* < 0.01 *versus* the control.

To assess *in vivo* the anti-inflammatory effects of MK-2, which was the strongest inhibitor of animal pols and strongest suppressor of TPA-induced inflammation and LPS-evoked TNF-α production among the VK_2_ and VK_3_ intermediates, we investigated the inhibitory activity of this compound against LPS-induced acute inflammation (Figure 6). Treatment with 250 μg/kg BW of LPS considerably increased the serum TNF-α level (5 ng/mL), and intraperitoneal injection of 100 mg/kg BW of MK-2 greatly decreased this LPS-induced TNF-α production by 83.2%. Thus, the *in vivo* data obtained in this mouse study showed the same trend as the data obtained from cultured mouse macrophage cells (Figures 4 and 5).

## 3. Discussion

VK comprises a group of 2-methyl-1,4-naphthoquinone derivatives. It is a hydrophobic (*i.e.*, insoluble in water) human vitamin. It is needed for the synthesis of proteins required for blood coagulation [31]. Normally, VK is produced by bacteria in the intestines, and dietary deficiency is extremely rare unless the intestines are badly damaged. VK is involved in the formation of calcium-binding groups in proteins [32,33]. Recently, DNA microarray was used to identify the effect of VK status on gene expression in the rat liver. The expression of genes involved in the acute inflammation response was enhanced in rats fed a VK-deficient diet relative to the control and VK-supplemented diet groups [34].

VK_1_ and VK_2_ are naturally occurring types of VK. VK_1_ is synthesized by plants and can be found in such foods as spinach, broccoli, lettuce, and soybeans. VK_2_ is primarily produced by bacteria in the anterior part of the gut and the intestines. The MK-4 and MK-7 forms of VK_2_ are found in meat, eggs, dairy and natto. MK-4 is synthesized by animal tissues, other forms of VK_2_ (mainly MK-7) are synthesized by bacteria during fermentation [35]. In natto 0% of VK is in the MK-4 form, and in cheese 2–7% is in this form [36]. VK_3_, on the other hand, is one of the many manmade versions of VK. Also called menadione, this yellowish, synthetic crystalline substance is converted into the active form of VK_2_ inside the animal body [37]. VK_2_ forms with 2–13 isoprene units, including MK-1, MK-2 and MK-3, have been found in human and animal tissues [37]. On the other hand, Booth reported that though VK_2_ derivatives are synthesized in the intestine, intestinal MKs are not believed to be the primary source of VK; VK_1_ is the primary dietary source; MK-4 and MK-7 are relatively minor sources in the average diet [38].

We have shown here that compounds that are intermediates between VK_2_ (MK-4) and VK_3_ potently inhibit the activity of mammalian pols, especially pol λ (Table 1 and Figure 2), and that these compounds prevented the inflammatory response *in vitro* and *in vivo* (Figures 3 to 6). In particular, MK-2 showed the strongest of these effects among MK-1 to MK-4; therefore, the length of the side chain of MK-*n* at the 3-position of VK_2_ must be very important for these inhibitory activities. As reported previously, the phenolic compound curcumin, which is a known anti-inflammatory agent, is a pol-λ-specific inhibitor [6,19,20]. Intriguingly, the principle molecular target of the VK_2_ and VK_3_ intermediates, such as MK-2, is also pol λ.

Eukaryotic cells reportedly contain 15 pol species belonging to four families: namely, family A (pols γ, θ, and ν), family B (pols α, δ, ɛ and ζ), family X (pols β, λ and μ, and TdT) and family Y (pols η, ι and κ, and REV1) [3,4]. Among the X family of pols, pol λ seems to work in a similar way to pol β [39]. Pol β is involved in the short-patch base excision repair (BER) pathway [40–43], as well as playing an essential role in neural development [44]. Recently, pol λ was found to possess 5′-deoxyribose-5-phosphate (dRP) lyase activity, but not apurinic/apyrimidinic (AP) lyase activity [45]. Pol λ is able to substitute for pol β during *in vitro* BER, suggesting that pol λ also participates in BER. Northern blot analysis indicated that transcripts of pol β are abundantly expressed in the testis, thymus and brain in rats [46], whereas pol λ is efficiently transcribed mostly in the testis [47]. Bertocci *et al.* reported that mice in which pol λ expression is knocked out are not only viable and fertile, but also display a normal hyper-mutation pattern [48].

As well as causing inflammation, TPA influences cell proliferation and has physiological effects on cells because it has tumor promoter activity [24]. Therefore, anti-inflammatory agents are expected to suppress DNA replication/repair/recombination in nuclei in relation to the action of TPA. Because pol λ is a repair/recombination-related pol [39], our finding—that the molecular target of VK_2_ and VK_3_ intermediates such as MK-2 is pol λ—is in good agreement with this expected mechanism of anti-inflammatory agents. The detail mechanism by which MK-2 that prevents mammalian pol λ inhibition could inhibit inflammation is unclear; therefore, to clarify the exact mechanism of the anti-inflammatory effect of MK-2, further study will be conducted.

We have investigated the inhibitory effect of the VK_2_ and VK_3_ intermediates on the activity of mammalian pols, which are responsible for DNA replication leading to cell proliferation and DNA repair/recombination, as well as the relationship between the degree of the suppression of LPS-evoked TNF-α production and anti-inflammatory activity. As a result, we found a positive correlation between the pol inhibitory activity and the anti-inflammatory activity.

## 4. Experimental Section

### 4.1. Materials

VK_2_ (MK-4) and VK_3_ were obtained from Sigma-Aldrich (St. Louis, MO, U.S.), and these structures are shown in Figure 1. Each compound was purified to more than 99% purity. Chemically synthesized DNA templates such as poly(dA) and nucleotides such as [^3^H]-deoxythymidine 5′-triphosphate (dTTP) (43 Ci/mmol) were purchased from GE Healthcare Bio-Sciences (Little Chalfont, U.K.). The oligo(dT)_18_ DNA primer was customized by Sigma-Aldrich Japan K.K. (Hokkaido, Japan). LPS was purchased from Sigma-Aldrich. For Western blot analysis, anti-NF-κB p65 antibody, anti-β-actin antibody and horseradish peroxidase-conjugated anti-rabbit IgG antibody (*i.e.*, secondary antibody) were obtained from Santa Cruz Biotechnology (Santa Cruz, CA, U.S.) and Thermo Scientific (Kanagawa, Japan), respectively. All other reagents were of analytical grade and purchased from Nacalai Tesque Inc. (Kyoto, Japan).

### 4.2. Preparation of the VK_2_ and VK_3_ Intermediates

The VK_2_ and VK_3_ derivatives (*i.e.*, MK-3, MK-2 and MK-1) were chemically synthesized according to a procedure reported by Mayer and Isler with slight modification [49]. The structures of these compounds were confirmed by comparison of the spectral data with the reported data, and are shown in Figure 1.

### 4.3. Pol and Other DNA Metabolic Enzyme Assays

Pols from mammals, a fish (cherry salmon), an insect (fruit fly) and a plant (cauliflower) were purified, and prokaryotic pols and other DNA metabolic enzymes, such as T7 RNA polymerase, T4 polynucleotide kinase and bovine deoxyribonuclease I, were purchased as described in our previous report [50]. The activities of all pols and other DNA metabolic enzymes were measured as described in previous reports [50–53].

The components of the pol assay were poly(dA)/oligo(dT)_18_ and dTTP as the DNA template-primer and 2′-deoxyribonucleoside 5′-triphosphate (dNTP) substrate, respectively. VK_2_, VK_3_, and their synthesized derivatives (*i.e.*, MK-3, MK-2 and MK-1) were dissolved in dimethyl sulfoxide (DMSO) at various concentrations and sonicated for 30 s. The sonicated samples (4 μL) were mixed with 16 μL of each pol enzyme (final amount, 0.05 units) in 50 mM Tris-HCl (pH 7.5) containing 1 mM dithiothreitol, 50% glycerol and 0.1 mM EDTA, and kept at 0 °C for 10 min. These inhibitor–enzyme mixtures (8 μL) were added to 16 μL of each standard enzyme reaction mixture (50 mM Tris-HCl [pH 7.5], 1 mM dithiothreitol, 1 mM MgCl_2_, 15% glycerol, 10 μM poly(dA)/oligo(dT)_18_ and 10 μM [^3^H]-dTTP), and incubation was carried out at 37 °C for 60 min, except for *Taq* pol, which was incubated at 74 °C for 60 min. Activity without the inhibitor was considered to be 100%, and the activity remaining at each concentration of inhibitor was determined relative to this value. One unit of pol activity was defined as the amount of enzyme that catalyzed the incorporation of 1 nmol dNTP (dTTP) into the synthetic DNA template-primer (poly(dA)/oligo(dT)_18_, A/T = 2/1) in 60 min at 37 °C under normal reaction conditions for each enzyme (scintillation counts: approximately 1 pmol of incorporated radioactive nucleotides = 100 cpm) [51,52].

### 4.4. Animal Experiments

All animal studies were performed according to the guidelines outlined in the “Care and Use of Laboratory Animals” of Kobe University. The animals were anesthetized with pentobarbital before undergoing cervical dislocation. Female 8-week-old C57BL/6 mice that had been bred in-house with free access to food and water were used for all experiments. All of the mice were maintained under a 12-h light/dark cycle and housed at a room temperature of 25 °C.

### 4.5. TPA-Induced Anti-Inflammatory Assay in Mouse

The mouse inflammatory test was performed according to Gschwendt’s method [53]. In brief, an acetone solution of the VK_2_ and VK_3_ intermediates (250 or 500 μg in 20 μL) or 20 μL of acetone as a vehicle control was applied to the inner part of the mouse ear. Thirty minutes after the test compound was applied, a TPA solution (0.5 μg/20 μL of acetone) was applied to the same part of the ear. To the other ear of the same mouse, methanol, followed by TPA solution, was applied as a control. After 7 h, a disk (6 mm diameter) was obtained from the ear and weighed. The inhibitory effect (IE) is presented as a ratio of the increase in weight of the ear disks:

IE=[(TPA only)−(tested compound plus TPA)]/[(TPA only)−(vehicle)]×100

### 4.6. Measurement of TNF-α Level in the Cell Culture Medium of Mouse Macrophages

A mouse macrophage cell line, RAW264.7, was obtained from American Type Culture Collection (ATCC) (Manassas, VA, U.S.). The cells were cultured in Eagle’s Minimum Essential Medium (MEM) supplemented with 4.5 g of glucose per liter plus 10% fetal calf serum, 5 mM L-glutamine, 50 units/mL penicillin and 50 units/mL streptomycin. The cells were cultured at 37 °C in standard medium in a humidified atmosphere of 5% CO_2_–95% air.

RAW264.7 cells were placed in a 12-well plate at 5 × 10^4^ cells/well and incubated for 24 h. The cells were pretreated with the VK_2_ and VK_3_ intermediates (final concentrations of 10 and 50 μM) for 30 min before the addition of 100 ng/mL of LPS. After stimulation with LPS for 24 h, the cell culture medium was collected to measure the amount of TNF-α secreted. The concentration of TNF-α in the culture medium was quantified by using a commercially available enzyme-linked immunosorbent assay (ELISA) development system (Bay Bioscience Co., Ltd., Kobe, Japan) in accordance with the manufacturer’s protocol.

### 4.7. Cell Treatment and Preparation of Nuclear Proteins

RAW264.7 cells on a 6-well plate at 5 × 10^5^ cells/well were incubated with 10 μM of MK-2 or DMSO (1 μL/mL) as a vehicle control for 30 min, followed by treatment with 100 ng/mL of LPS for 30 min. After treatment, cells were harvested with lysis buffer consisting of 10 mM Hepes (pH 7.9), 10 mM KCl, 1.5 mM MgCl_2_ and 0.5 mM DTT containing protease inhibitors (1 mM phenylmethylsulfonyl fluoride [PMSF], 5 μg/mL of leupeptin and 5 μg/mL of aprotinin) and phosphatase inhibitors (10 mM NaF and 1 mM Na_3_VO_4_) and stood on ice for 15 min with occasional mixing. The mixture was centrifuged at 1000 × g for 10 min at 4 °C. The precipitate was suspended in extraction buffer consisting of 20 mM Hepes (pH 7.6), 20% (v/v) glycerol, 500 mM NaCl, 1.5 mM MgCl_2_, 0.2 mM EDTA, 1.0 mM DTT and 0.1% (v/v) Nonidet P-40 containing the same protease and phosphatase inhibitors. The suspension was then gently mixed on a rotary device for 1 h at 4 °C before being centrifuged at 15,000 × g for 20 min at 4 °C. The resulting supernatant was used as a nuclear extract. The protein concentration was measured by using a bicinchoninic acid (BCA) assay kit in accordance with the manufacturer’s protocol. The nuclear proteins were subjected to Western blotting to evaluate the nuclear translocation of NF-κB.

### 4.8. Western Blotting

The nuclear proteins (30–50 μg of protein) were boiled in a quarter-volume of sample buffer (1 M Tris-HCl [pH 7.5], 640 mM 2-mercaptoethanol, 0.2% bromphenol blue, 4% SDS and 20% glycerol) and then separated on 10% SDS polyacrylamide gels. Each gel was then electroblotted onto a PVDF membrane. The membrane was blocked with 1% skimmed milk in Tris-buffered saline TBS-T (10 mM Tris-HCl, 100 mM NaCl and 0.5% Tween-20) and probed with anti-NF-κB p65 antibody (1:500) and anti-β-actin antibody (1:5000), before being reacted with horseradish peroxidase (HRP)-conjugated secondary antibody (1:5000 and 1:20,000, respectively). The protein-antibody complex was visualized with ChemiLumiONE (Nacalai Tesque, Kyoto, Japan) and detected using an Image Reader (LAS-3000 Imaging System, Fuji Photo Film, Tokyo, Japan). The intensity of each band was analyzed using ImageJ, which was developed at the National Institute of Health.

### 4.9. *In Vivo* LPS-Induced Inflammatory Experiment

Mice were intraperitoneally injected with 200 μL of MK-2 dissolved in corn oil at 100 mg/kg body weight (BW), or 200 μL of corn oil as a vehicle control. After 30 min, the mice were intraperitoneally injected with 200 μL of 250 μg/kg BW LPS dissolved in PBS or 200 μL of PBS as a vehicle control. After 1 h, the mice were killed, and blood samples were collected. The blood serum was separated by centrifugation at 15,000 × g for 10 min at 4 °C, and the TNF-α level in the serum was measured by ELISA.

### 4.10. Statistical Analysis

All data are expressed as the means ± SE of at least three independent determinations for each experiment. Statistical significance between each experimental group was analyzed using Student’s t-test, and a level of probability of 0.01 and 0.05 was used as the criterion of significance.

## 5. Conclusions

Our study is the first to demonstrate that MK-2, which is an intermediate between VK_2_ (MK-4) and VK_3_, potently inhibited the activity of animal pols, especially pol λ. MK-2 also suppressed mouse ear inflammation stimulated by TPA and reduced NF-κB activation and TNF-α production. The molecular mechanism that links the LPS-induced inflammatory response and anti-inflammatory activity in the model of TPA-induced ear edema is unknown. Because activated NF-κB has been observed in a model of TPA-induced ear edema [54], the anti-inflammatory effects of MK-2 may be, at least in part, dependent on the inhibition of NF-κB activation. Our study indicates that MK-2 is useful as an NF-κB inhibitor and may be a potent chemopreventive agent against inflammation.

## Figures and Tables

**Table 1.** IC_50_ values of MK-2 against the activities of various DNA polymerases and other DNA metabolic enzymes.

**Enzyme**	**IC_50_ Value of MK-2 (μM)**
• **Mammalian DNA Polymerases**	
**A-family DNA polymerase**	
Human DNA polymerase γ	68.8 ± 3.4
**B-family DNA polymerase**	
Calf DNA polymerase α	27.6 ± 1.6
Human DNA polymerase δ	29.1 ± 1.8
Human DNA polymerase ɛ	28.2 ± 1.6
**X-family DNA polymerase**	
Rat DNA polymerase β	31.0 ± 1.8
Human DNA polymerase λ	24.6 ± 1.4
Calf Terminal deoxynucleotidyl transferase	29.4 ± 1.7
**Y-family DNA polymerase**	
Human DNA polymerase η	37.8 ± 2.2
Mouse DNA polymerase ι	39.0 ± 2.3
Human DNA polymerase κ	35.3 ± 2.1

• **Fish DNA Polymerase**	
Cherry salmon DNA polymerase δ	32.5 ± 1.9

• **Insect DNA Polymerases**	
Fruit fly DNA polymerase α	34.0 ± 2.0
Fruit fly DNA polymerase δ	36.9 ± 2.2
Fruit fly DNA polymerase ɛ	36.7 ± 2.1

• **Plant DNA Polymerase**	
Cauliflower DNA polymerase α	>200

• **Prokaryotic DNA Polymerases**	
*E. coli* DNA polymerase I	>200
*Taq* DNA polymerase	>200
T4 DNA polymerase	>200

• **Other DNA Metabolic Enzymes**	
Calf primase of DNA polymerase α	>200
T7 RNA polymerase	>200
T4 polynucleotide kinase	>200
Bovine deoxyribonuclease I	>200
